# Diet specialization and brood parasitism in cuckoo species

**DOI:** 10.1002/ece3.6263

**Published:** 2020-04-16

**Authors:** Federico Morelli, Yanina Benedetti, Anders Pape Møller

**Affiliations:** ^1^ Faculty of Environmental Sciences Department of Applied Geoinformatics and Spatial Planning Czech University of Life Sciences Prague Czech Republic; ^2^ Faculty of Biological Sciences University of Zielona Góra Zielona Góra Poland; ^3^ Ecologie Systématique Evolution Université Paris‐Sud CNRS Université Saclay Orsay Cedex France

**Keywords:** brood parasitism, cuckoos, diet specialization, functional traits, generalist, phylogenetic signal

## Abstract

1. Brood parasitism is a breeding strategy adopted by many species of cuckoos across the world. This breeding strategy influences the evolution of life histories of brood parasite species.
2. In this study, we tested whether the degree on diet specialization is related to the breeding strategy in cuckoo species, by comparing brood parasite and nonparasite species. We measured the gradient of diet specialization of cuckoos, by calculating the Gini coefficient, an index of inequality, on the multiple traits describing the diet of species. The Gini coefficient is a measure of statistical dispersion on a scale between 0 and 1, reflecting a gradient from low to high specialization, respectively. First, we tested the strength of the phylogenetic signal of diet specialization index among cuckoo species worldwide. Then, we ran phylogenetic generalized least square (PGLS) models to compare diet specialization, distribution range, and body mass of parasitic and nonparasitic cuckoo species, considering the phylogenetic signal of data.
3. After adjusting for the phylogenetic signal of the data and considering both, species distribution range and species body mass, brood parasitic cuckoos were characterized by higher diet specialization than nonbrood parasitic species. Brood parasitic species were also characterized by a larger breeding distribution range than nonparasitic species.
4. The findings of this study provide an additional understanding of the cuckoos’ ecology, relating diet and breeding strategies, information that could be important in conservation ecology.

## Graphical abstract

A diet specialization index for cuckoo species based on functional traits was estimated. We tested whether the degree on diet specialization is related to the breeding strategy. Brood parasitic shown a higher diet specialization than nonbrood parasitic species.

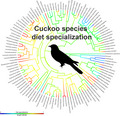

## 1 INTRODUCTION

The family Cuculidae, the unique taxon in the order Cuculiformes, is represented by 144 species of birds from 38 genera, with a cosmopolitan distribution except for polar regions, and the majority of species inhabiting the tropics (Davies, 2000; Erritzøe, Mann, Brammer, & Fuller, 2012). Many cuckoo species are characterized by a reproductive strategy known as brood parasitism (Davies, 2000). Brood parasitic species lay their eggs in the nests of other birds, sparing themselves the expense of rearing their own young (Erritzøe et al., 2012; Medina, Langmore, & Norris, 2016). Parasitism strongly influences the evolution of life histories of species (Krüger, 2007; Møller, 1997). Specifically, brood parasitism offers many advantages to species adopting such a strategy. The most obvious advantage is to reduce the overall costs of reproduction (Soler, 1999), and the capacity to spread nesting failure by laying eggs in several nests (Ducatez, 2014; Krüger & Davies, 2002; Payne, 2005). However, some studies also documented an increase in the probability of success in offspring from parasitic compared to nonparasitic species (Soler, 1999). Furthermore, brood parasite species could be less exposed to risk of extinction, having more stable population trends than species with parental care (Ducatez, 2014). The main reasons for that are related to the capacity to spread nesting failure risks associated with environmental changes among different host species, making brood parasites virtually more suitable to face global changes (Ducatez, 2014). Additionally, brood parasitism could also affect the foraging ecology of species evolved with such a breeding strategy. Food and nutrient limitation can have negative effects on the survival, reproduction, and fitness of individuals (Maklakov et al., 2008; Partridge & Harvey, 1985). Considering that brood parasitic cuckoos are virtually exempt from the costs of investment in parental care, we can expect to find differences in the diet and foraging strategies between parasitic and nonparasitic species. Such differences could be reflected in terms of a gradient of diet specialization. Specifically, we expected that parasitic species could be more specialized on some dietary items, as they would have no pressure to raise their offspring. In contrast, species with parental care should be characterized by a broader dietary preference, necessary to better guarantee adequate nutrients for the brood.

The strict ecological specialists species are typically defined as those occupying a relatively narrow niche or a restricted range of habitats, or using only a portion of the available resources in the habitat (Clavel, Julliard, & Devictor, 2011). The species defined as ecologically generalists, in contrast, are species able to thrive on a wide variety of environmental conditions, exploiting a large variety of available resources across space or time (Ducatez, Clavel, & Lefebvre, 2015; Irschick, Dyer, & Sherry, 2005). Some studies linked the degree of specialization with the extinction risk, suggesting that specialist species could be more exposed to extinction, due to a lower capacity of responding to environmental changes (Colles, Liow, & Prinzing, 2009; McKinney, 1997; Vázquez & Simberloff, 2002). Specialization can be considered a syndrome‐like modification of the entire phenotype, making exploitation of specific resources more efficient (Devictor et al., 2010). On the other hand, ecological “generalism” can be related to the aptitude to colonize new territories, exploiting new resources (Barnagaud, Devictor, Jiguet, & Archaux, 2011), and for this reason be associated with the global distribution range of species. From this point of view, brood parasitism of cuckoo species can be considered a type of ecological specialization (Krüger & Davies, 2002), and then be associated with the overall distribution of the worldwide cuckoo species, depending on their breeding strategy. In this regard, we expect that species with broader distribution ranges can also be characterized by higher variability in the diet (lower diet specialization) than species with narrower distribution ranges, in line with the niche variation hypothesis (Bolnick, Svanbäck, Araújo, & Persson, 2007; Maldonado, Bozinovic, Newsome, & Sabat, 2017).

In this study, we tested whether the degree of diet specialization is related to the breeding strategy, distribution range and body mass of cuckoo species. First, we developed an index of diet specialization based on a set of ecological characteristics describing the diet preferences of the species. Then, we tested the phylogenetic distribution of the diet specialization index through the phylogeny of cuckoo species across the world, by calculating the phylogenetic signal. Finally, we ran a statistical model focusing on the potential associations between diet specialization index, distribution range, body mass, and brood parasitism in cuckoo species taking phylogenetic similarity among related taxa into account.

## 2 METHODS

### 2.1 Diet specialization and distribution range of cuckoo species worldwide

In order to estimate the degree of diet specialization in cuckoo species, we followed the same methodology introduced by Morelli, Benedetti, Møller, and Fuller (2019). Briefly, we used a set of functional traits of bird species of the world, focusing on the different types of diet, provided in a recent publication (Wilman et al., 2014). The list of species traits focusing on diet type is given in Table 1 and is based in semi‐quantitative information. All variables are expressed as a percentage from 0 to 100 describing the preference in overall diet of the species. We estimated the degree of diet specialization using the Gini index of inequality (Gini, 1921). This index is based on the Gini coefficient, a measurement of statistical dispersion on a scale between 0 and 1, representing low to high specialization, respectively (Colwell, 2011). This measure, developed in 1921 by the statistician Corrado Gini, is a single measure of inequality (Gastwirh, 1972; Gini, 1921). This index is often used to assess economic inequalities (Lerman & Yitzhaki, 1984), and was also adopted in some ecological studies for example to measure the evenness of coverage of protected areas among habitat types (Barr et al., 2011).

**TABLE 1:** Diet type of cuckoo species distributed worldwide used for estimation of the diet specialization index. All variables are expressed as a percentage from 0 to 100 describing the preference in the overall diet of the cuckoo species. The data are based on the semi‐quantitative information about relative importance of different categories of the diet (Wilman et al., 2014)

Variable	Diet category	Details
1	Invertebrates	Percentage of the item in the overall diet (%)
2	Vertebrates (endotherm)	Percentage of the item in the overall diet (%)
3	Vertebrates (ectotherm)	Percentage of the item in the overall diet (%)
4	Vertebrates (fish)	Percentage of the item in the overall diet (%)
5	Vertebrates (unknown)	Percentage of the item in the overall diet (%)
6	Scavenger	Percentage of the item in the overall diet (%)
7	Frugivore	Percentage of the item in the overall diet (%)
8	Nectarivore	Percentage of the item in the overall diet (%)
9	Granivore	Percentage of the item in the overall diet (%)
10	Folivore	Percentage of the item in the overall diet (%)

The Gini coefficient is estimated with the following formula:

$$G=\frac{\sum_{i=1}^{n}\sum_{j=1}^{n}\left[x_{i}-x_{j}\right]}{2n^{2}\bar{x}}$$

where “x” is an observed value, “n” is the number of values observed and “ $\overset{-}{x}$ ” is the mean value.

When applied to the table describing the different types of diet of species (diet specialization), if every variable in a group has the same value or weight, the index would equal 0, indicating the maximum generalism. In contrast, the Gini coefficient would equal 1, indicating perfect inequality (high diet specialization), when a species has a diet entirely composed of a single value or trait. Applying this procedure, we calculated diet specialism for each cuckoo species, regarding the complete set of avian species in the world (Wilman et al., 2014). The Gini coefficient for diet specialization was calculated using the package “DescTools” for R (Signorell & mult. al., 2019). Finally, the index was standardized between 0 (generalist species) to 1 (specialist species).

The worldwide distribution range of cuckoo species was obtained from the literature (Davies, 2000) and from the section “data zone” in the BirdLife website (http://datazone.birdlife.org/). The data on distribution range refer to the extent of occurrence of breeding/resident of each cuckoo species and is provided in square kilometers (km^2^) (IUCN & BirdLife International 2017). Additionally, we recorded overall body mass for each cuckoo species from the same publication used for the diet traits (Wilman et al., 2014).

### 2.2 Phylogenetic signal of specialization and phylogenetic generalized least squares (PGLS) model

The phylogenetic signal is defined as the tendency for related species to resemble each other, more than they resemble species drawn at random from a phylogenetic tree (Blomberg, Garland, & Ives, 2003), because all organisms descend from common ancestors and hence are related in a hierarchical fashion (Futuyma & Agrawal, 2009). As a consequence, a high phylogenetic signal suggests that species traits are more similar in close relatives than distant relatives, while the opposite (low phylogenetic signal) indicate that a trait is more similar in distant than close relatives or randomly distributed across a phylogeny (Kamilar & Cooper, 2013). The phylogenetic signal (Blomberg & Garland, 2003) in diet specialization was estimated by means of Blomberg's K statistic (Blomberg et al., 2003). When K approaches 1, trait evolution follows a mode of evolution that is consistent with Brownian motion, and if K > 1 close relatives are more similar than expected under Brownian motion, while if K < 1 closely related species are less similar than expected (Blomberg et al., 2003). Blomberg's K was estimated using the “phylosig” command of the “phytools” package for R (Revell, 2012).

Data on bird species cannot be treated as independent sampling units in comparative analyses, because species are evolutionarily related (Harvey & Purvis, 1991). Therefore, we modelled interspecific variation in diet specialism index across a phylogeny, obtaining the phylogenetic relationships among cuckoo species from “www.birdtree.org”. We downloaded 1,000 phylogenetic trees from the backbone tree based on Ericson et al. (2006) for the 119 cuckoo species that were the focus of this study (ESM, Table S1). The consensus tree was obtained applying the 50% majority rule (i.e., the proportion of a split to be present in all trees) (Rubolini, Liker, Garamszegi, Møller, & Saino, 2015). In order to manage phylogenetic trees, we used the following R packages: “ape” (Paradis, Claude, & Strimmer, 2004), “phangorn” (Schliep, 2011) and “Rphylip” (Revell & Chamberlain, 2014).

Phylogenetic regression of the diet specialization index on the breeding distribution range, body mass and parasitism (brood parasitic/ nonparasitic species) was carried out using the “pgls” command of the “caper” package for R. A test of variance inflation factor (VIF) of candidate model was applied to check for potential multi‐collinearity issues among predictors, using the “car” package for R (Fox & Weisberg, 2019). Standardized regression coefficients (beta) were obtained in PGLS models, in order to compare the magnitude of the effect among predictors (i.e., analyses were carried out with standardized variables, so that their averages are zero and variances are 1). We added the phylogenetic information on cuckoo species summarizing the tree set into a single consensus tree, which was incorporated as a phylogenetic hypothesis in the statistical model (Rubolini et al., 2015). A second set of phylogenetic regression was run comparing the breeding distribution range and then the body mass between brood parasitic and nonparasitic species of cuckoos. Both variables were modelled separately. We obtained the regression coefficients for the models, standard errors and 95% confidence intervals of regression coefficients (Burnham & Anderson, 2002).

All statistical tests were performed with R software version 3.6.0 (R Development Core Team 2019).

## 3 RESULTS

Diet specialization of 119 cuckoo species distributed worldwide (67 nonparasitic and 52 brood parasitic species, ESM, Table S1) was determined by estimating the Gini coefficient for the food preferences (Table 1).

Diet specialization ranged between a minimum of 0.116 and a maximum of 1 (diet specialist species) (ESM, Table S1). A fraction of 37% of the cuckoo species were classified as diet specialist species (45 from the total 119 cuckoo species). Within the diet specialists, approximately 58% of the species were brood parasites, while the remaining 42% were nonparasitic cuckoo species (ESM, Table S1). The four most diet generalist cuckoo species in the world were *Centropus sinensis*, *Geococcyx californianus*, *Crotophaga major* and *Crotophaga ani*, species with a distribution range average of 15,332,500 km^2^ (max: 21,700,000, min: 4,530,000) and a mean body mass of approximately 229 g (max: 376, min: 110.09) (ESM, Table S1).

**FIGURE 1:**
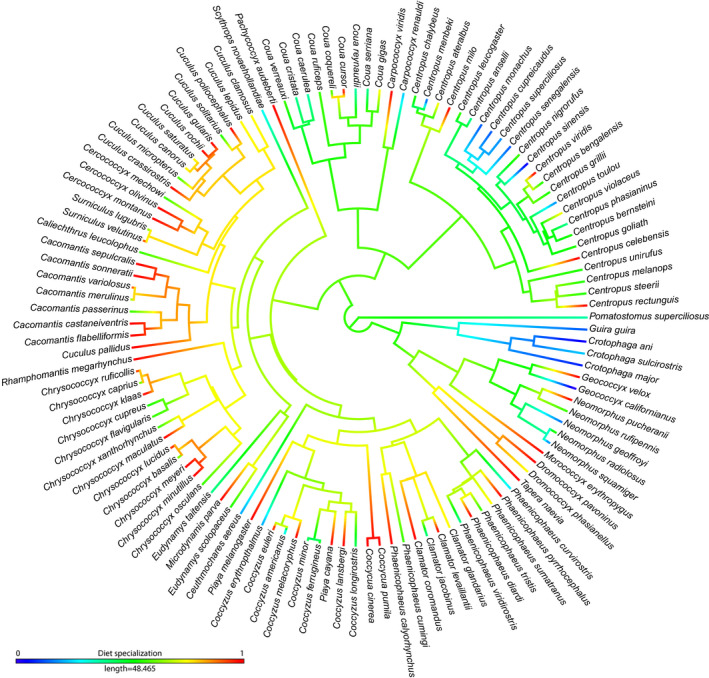
Dendrogram representing diet specialization in a colored gradient from generalist (dark blue) to specialist species (red). Tips represent the avian phylogeny of the 118 cuckoo species that are the focus of this study

Diet specialization showed a significant phylogenetic signal, with species being less similar than expected according to their phylogenetic relatedness, under a Brownian motion model (Blomberg's K statistic = 0.89, *p* = .008). However, diet specialist cuckoos occur in many different tips of the avian phylogeny of the 119 species that are the focus of this study (Figure 1).

**FIGURE 2:**
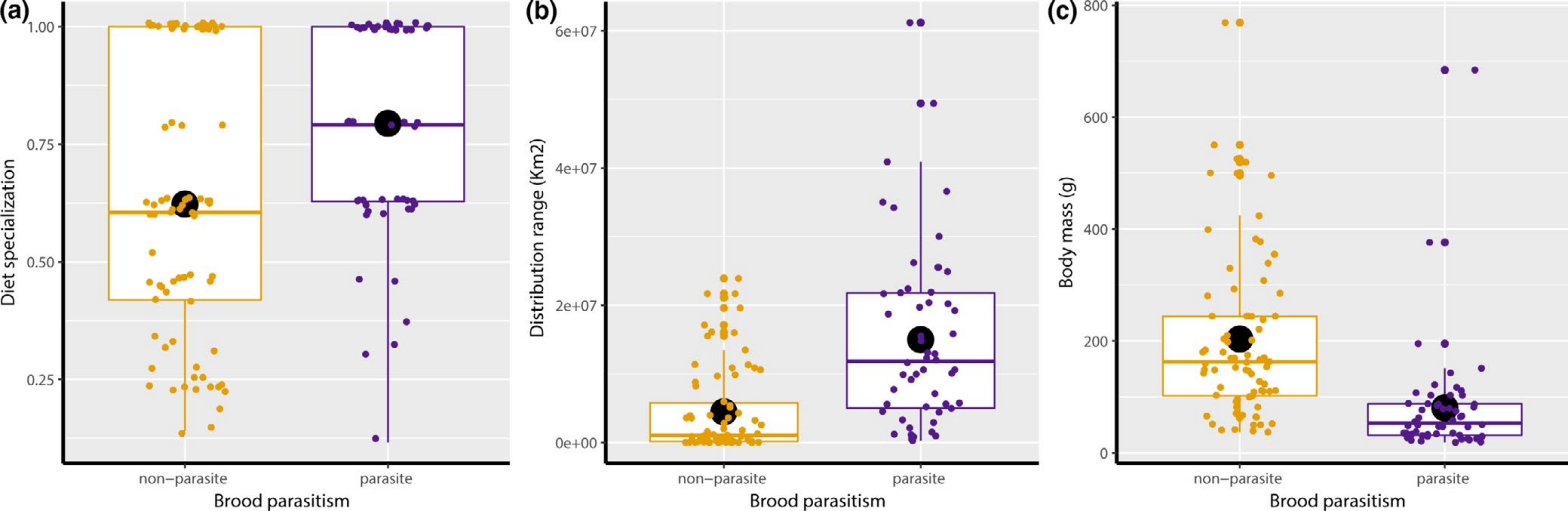
Diet specialization (a), distribution range (b) and body mass (c) of parasitic and nonparasitic species of Cuculidae. The box plots show medians, average values (black circle), quartiles, 5‐ and 95‐percentiles, and extreme values

The three predictors (brood parasitism, distribution range, and body mass) were modelled together because VIF was lower than 2 (1.47, 1.27 and 1.26, respectively). The results of the first model indicate that diet specialization is slightly positive and significantly associated with the distribution range of the species, while it is not associated with body mass of cuckoos (Table 2). Overall, brood parasitic cuckoos were characterized by a higher diet specialization than nonbrood parasitic species, and these differences were statistically significant (Table 2, Figure 2). Furthermore, brood parasitic cuckoos were characterized by a larger breeding range distribution than nonparasitic species (Table 3, Figure 2), while body mass differences highlighted in Figure 2 were not statistically significant when considering the phylogenetic signal of the data (Table 3).

**TABLE 2:** Results of Phylogenetic Generalized Least Squares (PGLS) model accounting for variation in diet specialization regressed on breeding distribution range, parasitism and body mass of cuckoo species across the world. The table shows estimates, standard error (SE), *t* statistic, and *P* values

Predictors	Estimate	*SE*	*t*	*P*
Intercept	0.593	0.301	1.965	.052
Parasitism (brood parasite)	0.184	0.065	2.816	.006
Distribution range	2e−6	1e−6	−3.155	.002
Body mass	−9e−5	3e−5	−0.328	.743

**TABLE 3:** Results of Phylogenetic Generalized Least Squares (PGLS) model accounting for variation in (A) breeding distribution range and (B) body mass between brood parasitic and nonparasitic cuckoo species across the world. The table shows estimates, standard error (SE), *t* statistic, and *P* values

Predictors	Estimate	*SE*	*t*	*P*
(A)				
Intercept	0.248	0.209	1.185	.238
Distribution range	2e−6	1e−6	2.774	.006
(B)				
Intercept	0.269	0.219	1.228	.222
Body mass	6e−5	2e−5	0.272	.786

## 4 DISCUSSION

The breeding strategy of brood parasitism has evolved several times in birds (Rothstein, 1990), and, from an evolutionary perspective, brood parasitic species seem to have evolved from nonparasitic species (Lanyon, 1992). Therefore, parasitism strongly influences the evolution of life histories of species, based on the condition of many of their ecological characteristics (Ducatez, 2014; Soler, 1999; Thomas, Guégan, Michalakis, & Renaud, 2000). In a previous study, we provided evidence that brood parasitic cuckoos are not more unique in terms of evolutionary distinctiveness than nonparasitic species (Morelli, Benedetti, Møller, Liang, & Carrascal, 2018). In the same study, focusing on parasitic cuckoos, we found that host specialist cuckoos are more evolutionarily unique than more host generalist species (e.g., Common cuckoo *Cuculus canorus*) (Morelli et al., 2018). Finally, we found a positive association between the number of host species (host range) and the area of distribution of parasitic cuckoos, suggesting a passive sampling of hosts by parasitic species as the breeding range broadens (Morelli et al., 2018).

The main findings of this study suggest that brood parasitic cuckoos are more diet specialist and more widely distributed than nonparasitic cuckoos. Results of the first model confirmed that, overall, brood parasitic species tend to exploit the narrowest range of food compared to nonparasitic species, therefore, achieving high values for the diet specialization index. Additionally, the results indicate also that diet specialization in cuckoos was slightly positively related to distribution range of the species. This fact is interesting, because it contradicts the expectation suggested by the niche variation hypothesis (Maldonado et al., 2017). On the other hand, the degree of diet specialization was not associated with the body mass of cuckoos.

In macroecology, ecological specialist or habitat specialist species are often associated with smaller distribution ranges than more generalist species (Williams et al., 2009). For this reason, ecologically specialist species also tend to be linked with lower response capacities when facing climate or environmental changes, making such species prone to higher extinction risks (Colles et al., 2009). From this point of view, our results linking a high diet specialization to larger distribution ranges in cuckoo species could appear to be slightly contrasting. We expected that more diet specialist cuckoos (as brood parasitic species are) should potentially be more exposed to extinction risks, and probably occupying smaller distribution ranges. In this study, we found that such species were characterized by a larger distribution range than nonparasitic species (which tend to be less diet specialists than brood parasitic species). This result should also be combined with the results of the second model that indicated a positive association between distribution range and brood parasitism, suggesting that brood parasitic species have overall large distribution ranges than nonbrood parasitic species. Therefore, we can speculate that brood parasitic cuckoos are successfully occupying worldwide larger distribution areas than nonparasitic species, occupying a wide variety of environments. Cuckoo habitat requirements are briefly defined as the source of food (mainly insects; Erritzøe et al., 2012; Payne, 2005) and a place to breed (for nonparasitic species) or presence of potential host species (for brood parasitic species) (Krüger, Sorenson & Davies 2009). The differences found in this study could be associated with the fact that many nonparasitic cuckoos are insular species or endemic species of small areas (e.g., the nine species of coua *Coua gigas*, *Coua serriana*, *Coua reynaudii*, *Coua cursor*, *Coua coquereli*, *Coua ruficeps*, *Coua caerulea*, *Coua cristata*, *Coua verreauxi* which are all endemic to Madagascar) (Erritzøe et al., 2012).

The expectation for a correlation between body mass and diet specialization in cuckoos was made by considering that similar associations between diet diversity or size of dietary items and body mass of species were already demonstrated for different vertebrates (Pineda‐Munoz, Evans, & Alroy, 2016; Sam, Koane, Jeppy, Sykorova, & Novotny, 2017). Basically, the body mass of individuals determines their energetic requirements constraining its diet (Jetz, Carbone, Fulford, & Brown, 2004). However, in bird species, the association between diet or foraging strategy and body mass is less clear, and often it is not linear (Olsen, 2015). In our study, the differences comparing body mass of birds with brood parasitic and nonparasitic species were not statistically significant, when considering the phylogenetic signal of data. Here, we highlight this observation even if the mean values were slightly different (mean = 79 g for brood parasitic while mean = 211 g for nonparasitic species), when considering the range of these values, the differences seem to be smaller (min = 19 to a max = 684 g for brood parasitic, while min = 37 to a max = 769 g for nonparasitic species).

The fact that brood parasitic cuckoos seem to be characterized by a relatively higher degree of diet specialization than nonparasitic cuckoos, confirms one of our expectations. One possibility could be that brood parasitic species, being virtually exempt from the costs of investment in parental care, are able to explore a different set of diet items (even narrow) than species with parental care. Under this hypothesis, cuckoos without parental care could become more diet “specialist”, because they are not subject to the energetic requirements inherent to support a brood. In contrast, species with parental care must be more “generalists” in terms of diet because they are constrained in obtaining enough energy for supporting brood development. Briefly, because the effort to rear their young is considerable, parents should be constrained to take energy from a wider set of prey or food. In other words, the lifestyle of brood parasite cuckoos could be significantly less energy demanding than for species with parental care, which could be reflected in a greater specialization also potentially reducing the intensity of interspecific competition.

However, this interpretation presents many frailties. The most important: Diet specialist species are not necessarily characterized by a diet of lower energetic content than generalist species (Bell, 1990; Cramp & Perrins, 1994). For example, some bird species which only select insects as prey items can provide an overall large amount of energy for feeding the brood than species providing a combination of seeds, fruits, and insects (Garvey & Whiles, 2019). In the case of cuckoo species, we verified that differences in the mean values of diet specialization between brood parasitic and nonparasitic species are mainly associated with an unbalanced number of generalist species between parasitic and nonparasitic cuckoos. Considering the 49 cuckoo species classified as diet specialists following our diet specialization index (species with Gini coefficient = 1), 47 are species which only use insects as food (25 brood parasitic and 22 nonparasitic cuckoos), while the remaining 2 species exclusively use fruits (1 brood parasitic and 1 nonparasitic cuckoos). However, when focusing on more diet generalist cuckoo species, the differences between brood parasitic and nonparasitic cuckoos were accentuated. Among the most diet generalist species (species with Gini coefficient <0.5), 32 were nonparasitic species, while only 7 species were brood parasitic cuckoos.

The analysis of diet specialism of cuckoo species also revealed that this trait showed a significant phylogenetic signal. However, considering the values of Blomberg's K statistic, we can assume that regarding diet specialization, closely related cuckoo species were less similar than expected under a Brownian motion model (Blomberg et al., 2003). Additionally, it is interesting to note how values of higher diet specialization were relatively uniformly distributed across different tips of the phylogenetic tree, and not clustered in specific phylogenetic branches (see Figure 1). This fact could reasonably be interpreted as diet specialization occurring a different number of times in the evolution of cuckoo species, as a response or adaptation to environmental characteristics or requirements, and this foraging strategy has evolved independently within the Cuculiformes order.

Finally, even considering that we focused on three different aspects of the ecology of cuckoos (parasitism, breeding distribution range, and body mass), aspects which can play a role in the foraging strategy of species, we prefer to be cautious about any interpretation linking these aspects as a causal association. Many other factors or variables were not explored in this study, and they could be significantly conditioning the level of diet specialization of birds, even much more than the three aspects that were the object of the present study (Garvey & Whiles, 2019; Terraube, Guixé, & Arroyo, 2014). Thus, we have preferred to follow a descriptive approach rather than trying to explain any causal relationship between diet specialization and brood parasitism of cuckoos. We simply compared the level of diet specialization between the two types of breeding strategies of cuckoos (parental care vs. parasitism), in association with the overall breeding distribution range of such species. The main importance of our findings is due to the fact that diet specialization could be assumed as an indicator of the potential conservation threat of species, if considering that more diet specialist species are expected to be more exposed to extinction risks (Colles et al., 2009). With this in mind, our intention was to assess whether more diet specialist cuckoos are brood parasitic or nonparasitic species, and also to assess if the distribution range of diet specialist cuckoos is smaller, equal or larger than for generalist cuckoo species.

A potential limitation on the diet specialization index used in this study could be the fact that the index provides a rather coarse characterization of the degree of diet specialization). The specialization index focuses only on how the different diet categories (invertebrates, plant–seeds, fruits, etc.) are evenly distributed within a given species' preference. To know whether a species primarily feeds on invertebrates, fruits, or both could be too general a perspective for judging whether there is true diet specialization or not, especially considering that diet specialization is a relative concept that can be used to compare different species or different individuals within a species (e.g., Bolnick et al. (2003), Woo, Elliott, Davidson, Gaston, and Davoren (2008)). However, it is difficult to obtain more detailed information on diet preferences of birds especially because of heterogeneity of sources and species‐specific studies. We argue that the use of a relatively uniform source of information is essential for guaranteeing the comparison among different species. Another potential drawback of the index used in this study could be related to the fact that the degree of diet specialization can change even within species. In some cases, species considered overall generalists could be characterized as strongly specialist individuals (Terraube et al., 2014).

Our findings provide new understanding of the ecology of cuckoo species, regarding the wide spatial distribution of more diet specialist species, as well as the association between exploitation of a wider set of food items and the breeding strategy of species. A first potential implication for this study could be to provide a tool (diet specialization index of cuckoos) to be combined with information on breeding distribution range, which can offer useful information on the assessment of overall conservation status of each species. Such data can be used to assess the potential capacities of cuckoos to respond when facing eventual environmental challenges or effects of climate change. Indications suggest that this information could be included in the assessment criteria currently used during the redaction of the IUCN Red List of threatened species (Morelli et al., 2019; Webb, 2008). An additional understanding of the main characteristics which make a species more susceptible to extinction is important, especially in conservation ecology.

## CONFLICT OF INTEREST

Authors of the manuscript declare that they have no conflict of interest.

## AUTHOR CONTRIBUTION

**Federico Morelli:** Conceptualization (lead); Data curation (equal); Formal analysis (equal); Methodology (equal); Project administration (equal); Resources (equal); Software (equal); Supervision (lead); Validation (equal); Visualization (equal); Writing‐original draft (lead); Writing‐review & editing (equal). **Yanina Benedetti:** Conceptualization (equal); Data curation (equal); Formal analysis (equal); Methodology (equal); Resources (equal); Software (equal); Validation (equal); Visualization (equal); Writing‐review & editing (equal). **Anders Møller:** Conceptualization (supporting); Data curation (supporting); Validation (supporting); Writing‐review & editing (supporting).

## Supporting information

Table S1

## Data Availability

The dataset generated during and/or analyzed during the current study is available in the Electronic Supplementary Material.

## References

[ece36263-bib-0001] Barnagaud, J. Y. , Devictor, V. , Jiguet, F. , & Archaux, F. (2011). When species become generalists: On‐going large‐scale changes in bird habitat specialization. Global Ecology and Biogeography, 20, 630–640.

[ece36263-bib-0002] Barr, L. M. , Pressey, R. L. , Fuller, R. A. , Segan, D. B. , McDonald‐Madden, E. , & Possingham, H. P. (2011). A new way to measure the world's protected area coverage (M. Perc, Ed.). PLoS ONE, 6(9), e24707). 21957458 10.1371/journal.pone.0024707 PMC3177831

[ece36263-bib-0003] Bell, G. P. (1990). Birds and mammals on an insect diet: A primer on diet composition analysis in relation to ecological energetics In Morrison M. L., Ralph C. J., Verner J., & Jehl J. R. Jr. (Eds.), Studies in avian biology, 13. Avian foraging: Theory, methodology, and applications (pp. 416–426). San Diego, CA: Cooper Ornithological Society.

[ece36263-bib-0004] Blomberg, S. P. , & Garland, T. (2003). Tempo and mode in evolution: Phylogenetic inertia, adaptation and comparative methods. Journal of Evolutionary Biology, 15, 899–910.

[ece36263-bib-0005] Blomberg, S. P. , Garland, T. J. , & Ives, A. R. (2003). Testing for phylogenetic signal in comparative data: Behavioral traits are more labile. Evolution, 57, 717–745. 12778543 10.1111/j.0014-3820.2003.tb00285.x

[ece36263-bib-0006] Bolnick, D. I. , Svanbäck, R. , Araújo, M. S. , & Persson, L. (2007). Comparative support for the niche variation hypothesis that more generalized populations also are more heterogeneous. Proceedings of the National Academy of Sciences of the United States of America, 104, 10075–10079. 17537912 10.1073/pnas.0703743104 PMC1891261

[ece36263-bib-0007] Bolnick, D. I. , Svanbäck, R. , Fordyce, J. A. , Yang, L. H. , Davis, J. M. , Hulsey, C. D. , & Forister, M. L. (2003). The ecology of individuals: Incidence and implications of individual specialization. American Naturalist, 161, 1–28. 10.1086/343878 12650459

[ece36263-bib-0008] Burnham, K. P. , & Anderson, D. R. (2002). Model selection and multimodel inference: A practical information‐theoretic approach, 2nd ed Verlag, New York, NY: Springer.

[ece36263-bib-0009] Clavel, J. , Julliard, R. , & Devictor, V. (2011). Worldwide decline of specialist species: Toward a global functional homogenization? Frontiers in Ecology and the Environment, 9, 222–228.

[ece36263-bib-0010] Colles, A. , Liow, L. H. , & Prinzing, A. (2009). Are specialists at risk under environmental change? Neoecological, paleoecological and phylogenetic approaches. Ecology Letters, 12, 849–863. 19580588 10.1111/j.1461-0248.2009.01336.x PMC2730552

[ece36263-bib-0011] Colwell, F. A. (2011). Measuring inequality. Oxford, UK: Oxford University Press.

[ece36263-bib-0012] Cramp, S. , & Perrins, C. (1994). The birds of the western palearctic. Oxford, UK: Oxford University Press.

[ece36263-bib-0013] Davies, N. B. (2000). Cuckoos, cowbirds and other cheats, 1st ed. London, UK: Poyser.

[ece36263-bib-0014] R Development Core Team. , (2019). R: A language and environment for statistical computing.

[ece36263-bib-0015] Devictor, V. , Clavel, J. , Julliard, R. , Lavergne, S. , Mouillot, D. , Thuiller, W. , … Mouquet, N. (2010). Defining and measuring ecological specialization. Journal of Applied Ecology, 47, 15–25.

[ece36263-bib-0016] Ducatez, S. (2014). Brood parasitism: a good strategy in our changing world? Proceedings of the Royal Society B: Biological Sciences, 281, 20132404. 10.1098/rspb.2013.2404 PMC4027383 24552836

[ece36263-bib-0017] Ducatez, S. , Clavel, J. , & Lefebvre, L. (2015). Ecological generalism and behavioural innovation in birds: Technical intelligence or the simple incorporation of new foods? Journal of Animal Ecology, 84, 79–89. 24910268 10.1111/1365-2656.12255

[ece36263-bib-0018] Ericson, P. G. P. , Anderson, C. L. , Britton, T. , Elzanowski, A. , Johansson, U. S. , Källersjö, M. , … Mayr, G. (2006). Diversification of Neoaves: Integration of molecular sequence data and fossils. Biology Letters, 2, 543–547. 17148284 10.1098/rsbl.2006.0523 PMC1834003

[ece36263-bib-0019] Erritzøe, J. , Mann, C. F. , Brammer, F. P. , & Fuller, R. A. (2012). Cuckoos of the World, 1st ed London, UK: Christopher Helm Publishers Ltd.

[ece36263-bib-0020] Fox, J. , & Weisberg, S. (2019). An R Companion to Applied Regression. Thirdn: SAGE Publications Inc, Thousand Oaks CA.

[ece36263-bib-0021] Futuyma, D. J. , & Agrawal, A. A. (2009). Evolutionary history and species interactions. Proceedings of the National Academy of Sciences of the United States of America, 106, 18043–18044. 19861552 10.1073/pnas.0910334106 PMC2775303

[ece36263-bib-0022] Garvey, J. E. , & Whiles, M. (2019). Trophic Ecology. Boca Raton, FL: CRC Press.

[ece36263-bib-0023] Gastwirh, J. (1972). The estimation of the lorenz curve and Gini index. The Review of Economics and Statistics, 54, 306–316.

[ece36263-bib-0024] Gini, C. (1921). Measurement of inequality and incomes. The Economic Journal, 31, 124–126.

[ece36263-bib-0025] Harvey, P. H. , & Purvis, A. (1991). Comparative methods for explaining adaptations. Nature, 351, 619–624. 2052088 10.1038/351619a0

[ece36263-bib-0026] Irschick, D. , Dyer, L. , & Sherry, T. W. (2005). Phylogenetic methodologies for studying specialization. Oikos, 110, 404–408.

[ece36263-bib-0027] IUCN & BirdLife International . (2017). IUCN 2017. The IUCN Red List of Threatened Species. Version 2016–3. www.iucnredlist.org

[ece36263-bib-0028] Jetz, W. , Carbone, C. , Fulford, J. , & Brown, J. H. (2004). The scaling of animal space use. Science, 306, 266–268. 15472074 10.1126/science.1102138

[ece36263-bib-0029] Kamilar, J. M. , & Cooper, N. (2013). Phylogenetic singal in primate behaviour, ecology anf life history. Philosophical Transactions of the Royal Society B, 368, 20120341. 10.1098/rstb.2012.0341 PMC3638444 23569289

[ece36263-bib-0030] Krüger, O. (2007). Cuckoos, cowbirds and hosts: Adaptations, trade‐offs and constraints. Philosophical Transactions of the Royal Society B, 362, 1873–1886. 10.1098/rstb.2006.1849 PMC2442387 17827098

[ece36263-bib-0031] Krüger, O. , & Davies, N. B. (2002). The evolution of cuckoo parasitism: a comparative analysis. Proceedings of the Royal Society of London. Series B: Biological Sciences, 269(1489), 375–381. 11886625 10.1098/rspb.2001.1887 PMC1690908

[ece36263-bib-0032] Krüger, O. , Sorenson, M. D. , & Davies, N. B. (2009). Does coevolution promote species richness in parasitic cuckoos? Proceedings of the Royal Society B: Biological Sciences, 276(1674), 3871–3879. 10.1098/rspb.2009.1142 PMC2817292 19692405

[ece36263-bib-0033] Lanyon, S. M. (1992). Interspecific brood parasitism in Balckbirds (Icterinae): A phylogenetic perspective. Science, 255, 77–79. 1553533 10.1126/science.1553533

[ece36263-bib-0034] Lerman, R. I. , & Yitzhaki, S. (1984). A note on the calculation and interpretation of the Gini index. Economic Letters, 15, 363–368.

[ece36263-bib-0035] Maklakov, A. A. , Simpson, S. J. , Zajitschek, F. , Hall, M. D. , Dessmann, J. , Clissold, F. , … Brooks, R. C. (2008). Sex‐specific fitness effects of nutrient intake on reproduction and lifespan. Current Biology, 18, 1062–1066. 18635354 10.1016/j.cub.2008.06.059

[ece36263-bib-0036] Maldonado, K. , Bozinovic, F. , Newsome, S. D. , & Sabat, P. (2017). Testing the niche variation hypothesis in a community of passerine birds. Ecology, 98, 903–908. 28170088 10.1002/ecy.1769

[ece36263-bib-0037] McKinney, M. (1997). Extinction vulnerability and selectivity: Combining ecological and paleontological views. Annual Review of Ecology and Systematics, 28, 495–516.

[ece36263-bib-0038] Medina, I. , Langmore, N. E. , & Norris, R. (2016). The evolution of host specialisation in avian brood parasites. Ecology Letters, 19, 1110–1118. 27417381 10.1111/ele.12649

[ece36263-bib-0039] Møller, A. P. (1997). Parasitism and the evolution of host life history In Clayton J. & Moore D. H. (Eds.), Host‐parasite evolution. General principles & avian models, (pp. 105–127.), Oxford, UK: University Press.

[ece36263-bib-0040] Morelli, F. , Benedetti, Y. , Møller, A. P. , & Fuller, R. A. (2019). Measuring avian specialization. Ecology and Evolution, 9, 8378–8386. 31380096 10.1002/ece3.5419 PMC6662403

[ece36263-bib-0041] Morelli, F. , Benedetti, Y. , Møller, A. P. , Liang, W. , & Carrascal, L. M. (2018). Cuckoos host range is associated positively with distribution range and negatively with evolutionary uniqueness. Journal of Animal Ecology, 87, 765–773. 29355941 10.1111/1365-2656.12797

[ece36263-bib-0042] Olsen, A. M. (2015). Exceptional avian herbivores: Multiple transitions toward herbivory in the bird order Anseriformes and its correlation with body mass. Ecology and Evolution, 5, 5016–5032. 26640679 10.1002/ece3.1787 PMC4662324

[ece36263-bib-0043] Paradis, E. , Claude, J. , & Strimmer, K. (2004). APE: Analyses of phylogenetics and evolution in R language. Bioinformatics, 20, 289–290. 14734327 10.1093/bioinformatics/btg412

[ece36263-bib-0044] Partridge, L. , & Harvey, P. H. (1985). Evolutionary biology: Costs of reproduction. Nature, 316, 20.

[ece36263-bib-0045] Payne, R. B. (2005). The cuckoos. New York: Oxford University Press.

[ece36263-bib-0046] Pineda‐Munoz, S. , Evans, A. R. , & Alroy, J. (2016). The relationship between diet and body mass in terrestrial mammals. Paleobiology, 42, 659–669.

[ece36263-bib-0047] Revell, L. J. (2012). phytools: An R package for phylogenetic comparative biology (and other things). Methods in Ecology and Evolution, 3, 217–223.

[ece36263-bib-0048] Revell, L. J. , & Chamberlain, S. A. (2014). Rphylip: an R interface for PHYLIP. Methods in Ecology and Evolution, 5(9), 976–981.

[ece36263-bib-0049] Rothstein, S. I. (1990). A model system for coevolution: Avian brood parasitism. Annual Review of Ecology and Systematics, 25, 481–508.

[ece36263-bib-0050] Rubolini, D. , Liker, A. , Garamszegi, L. Z. , Møller, A. P. , & Saino, N. (2015). Using the BirdTree.org website to obtain robust phylogenies for avian comparative studies: A primer. Current Zoology, 61, 959–965. 32256531 10.1093/czoolo/61.6.959 PMC7098689

[ece36263-bib-0051] Sam, K. , Koane, B. , Jeppy, S. , Sykorova, J. , & Novotny, V. (2017). Diet of land birds along an elevational gradient in Papua New Guinea. Scientific Reports, 7, 44018. 28276508 10.1038/srep44018 PMC5343654

[ece36263-bib-0052] Schliep, K. P. (2011). phangorn: Phylogenetic analysis in R. Bioinformatics, 27, 592–593. 21169378 10.1093/bioinformatics/btq706 PMC3035803

[ece36263-bib-0053] Signorell, A., (2019). DescTools: Tools for descriptive statistics. R package.

[ece36263-bib-0054] Soler, J. J. (1999). Brood parasites: The advantages of being a different species In Adams N. J., & Slotow R. H. (Eds.), Proc. 22 Int. Ornithol. Congr. (ed), (pp. 3098–3106). Johannesburg: BirdLife South Africa.

[ece36263-bib-0055] Terraube, J. , Guixé, D. , & Arroyo, B. (2014). Diet composition and foraging success in generalist predators: Are specialist individuals better foragers? Basic and Applied Ecology, 15, 616–624.

[ece36263-bib-0056] Thomas, F. , Guégan, J. F. , Michalakis, Y. , & Renaud, F. (2000). Parasites and host life‐history traits: Implications for community ecology and species co‐existence. International Journal for Parasitology, 30, 669–674. 10779584 10.1016/s0020-7519(00)00040-0

[ece36263-bib-0057] Vázquez, D. P. , & Simberloff, D. (2002). Ecological specialization and susceptibility to disturbance: Conjectures and refutations. The American Naturalist, 159, 606–623. 10.1086/339991 18707385

[ece36263-bib-0058] Webb, G. J. W. (2008). The dilemma of accuracy in IUCN Red List categories, as exemplified by hawksbill turtles Eretmochelys imbricata. Endangered Species Research, 6, 161–172.

[ece36263-bib-0059] Williams, S. E. , Williams, Y. M. , VanDerWal, J. , Isaac, J. L. , Shoo, L. P. , & Johnson, C. N. (2009). Ecological specialization and population size in a biodiversity hotspot: How rare species avoid extinction. Proceedings of the National Academy of Sciences, 17, 19737–19741. 10.1073/pnas.0901640106 PMC2780933 19897718

[ece36263-bib-0060] Wilman, H. , Belmaker, J. , Simpson, J. , de la Rosa, C. , Rivadeneira, M. M. , & Jetz, W. (2014). EltonTraits 1.0: Species‐level foraging attributes of the world’s birds and mammals. Ecology, 95, 2027.

[ece36263-bib-0061] Woo, K. J. , Elliott, K. H. , Davidson, M. , Gaston, A. J. , & Davoren, G. K. (2008). Individual specialization in diet by a generalist marine predator reflects specialization in foraging behaviour. Journal of Animal Ecology, 77, 1082–1091. 18624834 10.1111/j.1365-2656.2008.01429.x

