# Supplementary material for: Diet specialization and brood parasitism in cuckoo species
Source: Ecol Evol. 2020 Apr 16;10(11):5097–105. doi: 10.1002/ece3.6263 (PMC7297776; doi:10.1002/ece3.6263)
Supplement: Supplementary file 1 — Table S1 [file ECE3-10-5097-s001.docx]

**Electronic Supplementary Material - Diet specialization and brood parasitism in cuckoo species worldwide**

Table S1. List of Cuculidae species used in this study, parasite behavior, breeding distribution range and body mass. The breeding distribution range was obtained from IUCN (IUCN & BirdLife International 2017), while parasite behavior and body mass from published literature (Davies 2000; Wilman et al. 2014).

| **Species** | **Genus** | **Brood parasitism** | **Distribution range (km^2^)** | **Body mass (g)** | **Diet specialization** |
| --- | --- | --- | --- | --- | --- |
| *Centropus rectunguis* | Centropus | non-parasite | 2780000 | 238 | 1 |
| *Centropus steerii* | Centropus | non-parasite | 11700 | 198.43 | 0.605 |
| *Centropus melanops* | Centropus | non-parasite | 262000 | 221.2 | 0.605 |
| *Centropus unirufus* | Centropus | non-parasite | 214000 | 183.76 | 0.605 |
| *Centropus celebensis* | Centropus | non-parasite | 425000 | 244.36 | 1 |
| *Centropus goliath* | Centropus | non-parasite | 67300 | 499.42 | 0.605 |
| *Centropus bernsteini* | Centropus | non-parasite | 1010000 | 162.11 | 0.605 |
| *Centropus phasianinus* | Centropus | non-parasite | 9680000 | 381.98 | 0.465 |
| *Centropus violaceus* | Centropus | non-parasite | 127000 | 499.99 | 0.791 |
| *Centropus toulou* | Centropus | non-parasite | 854000 | 166.35 | 0.279 |
| *Centropus grillii* | Centropus | non-parasite | 17100000 | 122.88 | 0.605 |
| *Centropus bengalensis* | Centropus | non-parasite | 21100000 | 148.91 | 0.605 |
| *Centropus viridis* | Centropus | non-parasite | 711000 | 142 | 1 |
| *Centropus sinensis* | Centropus | non-parasite | 19600000 | 280.7 | 0.14 |
| *Centropus nigrorufus* | Centropus | non-parasite | 146000 | 244.36 | 0.628 |
| *Centropus senegalensis* | Centropus | non-parasite | 23900000 | 169 | 0.233 |
| *Centropus superciliosus* | Centropus | non-parasite | 16000000 | 148.45 | 0.279 |
| *Centropus cupreicaudus* | Centropus | non-parasite | 3210000 | 285.18 | 0.233 |
| *Centropus monachus* | Centropus | non-parasite | 9870000 | 201.31 | 0.233 |
| *Centropus anselli* | Centropus | non-parasite | 1580000 | 210 | 0.465 |
| *Centropus leucogaster* | Centropus | non-parasite | 3570000 | 307.81 | 0.628 |
| *Centropus milo* | Centropus | non-parasite | 46900 | 769 | 1 |
| *Centropus ateralbus* | Centropus | non-parasite | 143000 | 339 | 0.791 |
| *Centropus menbeki* | Centropus | non-parasite | 1330000 | 519 | 0.233 |
| *Centropus chalybeus* | Centropus | non-parasite | 3600 | 244.36 | 0.605 |
| *Carpococcyx renauldi* | Carpococcyx | non-parasite | 816000 | 550 | 0.326 |
| *Carpococcyx viridis* | Carpococcyx | non-parasite | 53800 | 525 | 1 |
| *Coua gigas* | Coua | non-parasite | 352000 | 424 | 0.791 |
| *Coua serriana* | Coua | non-parasite | 119000 | 293 | 0.465 |
| *Coua reynaudii* | Coua | non-parasite | 526000 | 153 | 0.442 |
| *Coua cursor* | Coua | non-parasite | 91500 | 110 | 1 |
| *Coua coquereli* | Coua | non-parasite | 351000 | 160 | 0.628 |
| *Coua ruficeps* | Coua | non-parasite | 133000 | 180 | 0.628 |
| *Coua caerulea* | Coua | non-parasite | 529000 | 244 | 0.442 |
| *Coua cristata* | Coua | non-parasite | 562000 | 147 | 0.465 |
| *Coua verreauxi* | Coua | non-parasite | 48600 | 203.75 | 0.628 |
| *Pachycoccyx audeberti* | Pachycoccyx | parasite | 15500000 | 102.85 | 1 |
| *Scythrops novaehollandiae* | Scythrops | parasite | 11600000 | 683.99 | 0.372 |
| *Cuculus clamosus* | Cuculus | parasite | 21800000 | 78.8 | 0.791 |
| *Cuculus lepidus* | Cuculus | parasite | 4950000 | 87 | 0.791 |
| *Cuculus poliocephalus* | Cuculus | parasite | 12300000 | 46.56 | 1 |
| *Cuculus solitarius* | Cuculus | parasite | 22400000 | 76.73 | 0.605 |
| *Cuculus gularis* | Cuculus | parasite | 21700000 | 103 | 1 |
| *Cuculus rochii* | Cuculus | parasite | 675000 | 62.7 | 1 |
| *Cuculus saturatus* | Cuculus | parasite | 36600000 | 87 | 0.791 |
| *Cuculus canorus* | Cuculus | parasite | 61200000 | 111.36 | 0.791 |
| *Cuculus micropterus* | Cuculus | parasite | 26200000 | 88.75 | 0.628 |
| *Cuculus crassirostris* | Cuculus | parasite | 271000 | 82.08 | 1 |
| *Cercococcyx mechowi* | Cercococcyx | parasite | 5600000 | 55.6 | 0.628 |
| *Cercococcyx olivinus* | Cercococcyx | parasite | 5200000 | 65.9 | 1 |
| *Cercococcyx montanus* | Cercococcyx | parasite | 2910000 | 58.2 | 1 |
| *Surniculus lugubris* | Surniculus | parasite | 7750000 | 29.7 | 0.628 |
| *Surniculus velutinus* | Surniculus | parasite | 828000 | 35.61 | 1 |
| *Caliechthrus leucolophus* | Caliechthrus | parasite | 954000 | 107.16 | 0.628 |
| *Cacomantis sepulcralis* | Cacomantis | parasite | 12000000 | 35.75 | 1 |
| *Cacomantis sonneratii* | Cacomantis | parasite | 14800000 | 33.8 | 1 |
| *Cacomantis variolosus* | Cacomantis | parasite | 24900000 | 35.75 | 1 |
| *Cacomantis merulinus* | Cacomantis | parasite | 12900000 | 25.3 | 0.628 |
| *Cacomantis passerinus* | Cacomantis | parasite | 3270000 | 25.3 | 0.628 |
| *Cacomantis castaneiventris* | Cacomantis | parasite | 2120000 | 33.2 | 1 |
| *Cacomantis flabelliformis* | Cacomantis | parasite | 19200000 | 49.8 | 1 |
| *Cuculus pallidus* | Cuculus | parasite | 10100000 | 87.69 | 1 |
| *Rhamphomantis megarhynchus* | Rhamphomantis | parasite | 1150000 | 31.5 | 1 |
| *Chrysococcyx ruficollis* | Chrysococcyx | parasite | 493000 | 21 | 1 |
| *Chrysococcyx caprius* | Chrysococcyx | parasite | 30000000 | 32.79 | 0.605 |
| *Chrysococcyx klaas* | Chrysococcyx | parasite | 25500000 | 27.37 | 1 |
| *Chrysococcyx cupreus* | Chrysococcyx | parasite | 21900000 | 37.7 | 0.605 |
| *Chrysococcyx flavigularis* | Chrysococcyx | parasite | 5560000 | 29.19 | 0.628 |
| *Chrysococcyx xanthorhynchus* | Chrysococcyx | parasite | 10600000 | 22.37 | 0.628 |
| *Chrysococcyx maculatus* | Chrysococcyx | parasite | 4400000 | 27.38 | 1 |
| *Chrysococcyx lucidus* | Chrysococcyx | parasite | 20200000 | 24.44 | 1 |
| *Chrysococcyx basalis* | Chrysococcyx | parasite | 10600000 | 23.12 | 0.628 |
| *Chrysococcyx meyeri* | Chrysococcyx | parasite | 25500000 | 19.42 | 1 |
| *Chrysococcyx minutillus* | Chrysococcyx | parasite | 15800000 | 18.6 | 1 |
| *Chrysococcyx osculans* | Chrysococcyx | parasite | 9150000 | 30.9 | 0.628 |
| *Eudynamys taitensis* | Eudynamys | parasite | 1520000 | 117 | 0.465 |
| *Microdynamis parva* | Microdynamis | parasite | 1170000 | 47.22 | 1 |
| *Eudynamys scolopaceus* | Eudynamys | parasite | 34200000 | 194.92 | 0.605 |
| *Ceuthmochares aereus* | Ceuthmochares | non-parasite | 8210000 | 65.8 | 0.302 |
| *Piaya melanogaster* | Piaya | non-parasite | 5380000 | 102.82 | 1 |
| *Coccyzus erythropthalmus* | Coccyzus | parasite | 7140000 | 50.9 | 0.302 |
| *Coccyzus euleri* | Coccyzus | non-parasite | 11300000 | 52.3 | 1 |
| *Coccyzus americanus* | Coccyzus | parasite | 35000000 | 64 | 0.326 |
| *Coccyzus melacoryphus* | Coccyzus | non-parasite | 21700000 | 49.71 | 1 |
| *Coccyzus minor* | Coccyzus | non-parasite | 897276 | 63.89 | 0.465 |
| *Coccyzus ferrugineus* | Coccyzus | non-parasite | 30 | 70 | 0.628 |
| *Piaya cayana* | Piaya | non-parasite | 16100000 | 101.98 | 1 |
| *Coccyzus lansbergi* | Coccyzus | non-parasite | 1050000 | 50.3 | 1 |
| *Coccyzus longirostris* | Saurothera | non-parasite | 125000 | 100 | 0.512 |
| *Coccycua cinerea* | Coccyzus | non-parasite | 2540000 | 41.04 | 1 |
| *Coccycua pumila* | Coccyzus | non-parasite | 1150000 | 37.1 | 1 |
| *Phaenicophaeus calyorhynchus* | Rhamphococcyx | non-parasite | 430000 | 111.35 | 1 |
| *Phaenicophaeus cumingi* | Lepidogrammus | non-parasite | 168000 | 170.46 | 0.628 |
| *Clamator coromandus* | Clamator | parasite | 13100000 | 74.59 | 1 |
| *Clamator jacobinus* | Clamator | parasite | 49400000 | 79.37 | 0.465 |
| *Clamator levaillantii* | Clamator | parasite | 20400000 | 122 | 1 |
| *Clamator glandarius* | Clamator | parasite | 40900000 | 143 | 0.791 |
| *Phaenicophaeus viridirostris* | Rhopodytes | non-parasite | 1780000 | 67 | 0.465 |
| *Phaenicophaeus diardi* | Rhopodytes | non-parasite | 3580000 | 62.1 | 1 |
| *Phaenicophaeus tristis* | Rhopodytes | non-parasite | 11400000 | 117 | 0.628 |
| *Phaenicophaeus sumatranus* | Rhopodytes | non-parasite | 3580000 | 91.94 | 0.791 |
| *Phaenicophaeus pyrrhocephalus* | Phaenicophaeus | non-parasite | 44300 | 111.35 | 1 |
| *Phaenicophaeus curvirostris* | Rhamphococcyx | non-parasite | 5160000 | 154 | 0.349 |
| *Tapera naevia* | Tapera | parasite | 19700000 | 48.42 | 1 |
| *Dromococcyx phasianellus* | Dromococcyx | parasite | 18700000 | 84.5 | 0.791 |
| *Dromococcyx pavoninus* | Dromococcyx | parasite | 9950000 | 46.4 | 1 |
| *Morococcyx erythropygus* | Morococcyx | non-parasite | 1240000 | 64.53 | 1 |
| *Neomorphus squamiger* | Neomorphus | non-parasite | 10900000 | 355.03 | 0.256 |
| *Neomorphus radiolosus* | Neomorphus | non-parasite | 58000 | 399 | 0.628 |
| *Neomorphus geoffroyi* | Neomorphus | non-parasite | 10900000 | 355.03 | 0.256 |
| *Neomorphus rufipennis* | Neomorphus | non-parasite | 915000 | 377.73 | 0.628 |
| *Neomorphus pucheranii* | Neomorphus | non-parasite | 1180000 | 330 | 1 |
| *Geococcyx californianus* | Geococcyx | parasite | 4530000 | 376 | 0.116 |
| *Geococcyx velox* | Geococcyx | non-parasite | 1990000 | 179.89 | 1 |
| *Crotophaga major* | Crotophaga | non-parasite | 15500000 | 148.25 | 0.186 |
| *Crotophaga sulcirostris* | Crotophaga | non-parasite | 13500000 | 82.04 | 0.326 |
| *Crotophaga ani* | Crotophaga | non-parasite | 21700000 | 110.09 | 0.14 |
| *Guira guira* | Guira | non-parasite | 10600000 | 141 | 0.233 |
| *Pomatostomus superciliosus* | Dasylophus | non-parasite | 5930000 | 41.53 | 0.442 |
